# The Scalp Time-Varying Networks of N170: Reference, Latency, and Information Flow

**DOI:** 10.3389/fnins.2018.00250

**Published:** 2018-04-18

**Authors:** Yin Tian, Wei Xu, Huiling Zhang, Kin Y. Tam, Haiyong Zhang, Li Yang, Zhangyong Li, Yu Pang

**Affiliations:** ^1^Chongqing Key Laboratory of Photoelectronic Information Sensing and Transmitting Technology, Chongqing High School Innovation Team of Architecture and Core Technologies of Smart Medical System, Bio-information College, Chongqing University of Posts and Telecommunications, Chongqing, China; ^2^Faculty of Health Sciences, University of Macau, Taipa, China

**Keywords:** N170, time-varying network, REST, AR, latency

## Abstract

Using the scalp time-varying network method, the present study is the first to investigate the temporal influence of the reference on N170, a negative event-related potential component (ERP) appeared about 170 ms that is elicited by facial recognition, in the network levels. Two kinds of scalp electroencephalogram (EEG) references, namely, AR (average of all recording channels) and reference electrode standardization technique (REST), were comparatively investigated via the time-varying processing of N170. Results showed that the latency and amplitude of N170 were significantly different between REST and AR, with the former being earlier and smaller. In particular, the information flow from right temporal-parietal P8 to left P7 in the time-varying network was earlier in REST than that in AR, and this phenomenon was reproduced by simulation, in which the performance of REST was closer to the true case at source level. These findings indicate that reference plays a crucial role in ERP data interpretation, and importantly, the newly developed approximate zero-reference REST would be a superior choice for precise evaluation of the scalp spatio-temporal changes relating to various cognitive events.

## Introduction

For scalp EEG/ERP data, the choice of reference is a critical issue for not only the ERP amplitude (Yao et al., 2005), the difference between two ERPs (Tian and Yao, 2013), but also the brain network topology (Yao et al., 2005; Qin et al., 2010; Thatcher, 2012). In fact, only the voltage differences between two points can be measured (Geselowitz, 1998). However, due to the lack of a neutral (or zero) point on the human body surface, all of the current recording references, such as the mean mastoid reference (MM) and the vertex reference (Cz), might unavoidably lead to some unknown false fluctuations that destroy the genuine EEG information (Yao, 2001; Zhai and Yao, 2004; Yao et al., 2007; Kayser and Tenke, 2010; Nunez, 2010; Qin et al., 2010; Tian and Yao, 2013). For average reference (AR), though it is the most widely used re-reference in current practice, its value is not the ideal zero reference not only due to the insufficient coverage, but also due to the non-spherical shape of our human head (Yao, 2017). As a point at infinity is theoretical far from the brain sources and has an ideal zero or neutral potential, a reference electrode standardization technique (REST) was proposed to mathematically re-reference the EEG recordings to infinity to get a zero reference (Yao, 2001), and its efficiency was repeatedly confirmed by the following studies (Marzetti et al., 2007; Tian and Yao, 2013; Chella et al., 2017). The details about REST were shown in Appendix 1. In this work, REST will be firstly used comparatively to AR to evaluate the potential effects on N170, which is an event-related potential elicited by face recognition (Bentin et al., 1996; Itier and Taylor, 2004).

Face recognition is an important ability in human daily communication. Previous electrophysiological studies found that pictures of faces elicited a larger scalp event-related potential (ERP) of negative amplitude peaking around 150–200 ms than other object categories (Bentin et al., 1996; Itier and Taylor, 2004). This early visual first negative component (N1) appeared at about 170 ms following face stimulus onset, which is termed as N170 (Bentin et al., 1996). The N170 is recorded at bilateral temporal-parietal electrode sites and is functionally associated with stages of face-specific structural encoding and/or faces detection (Bentin et al., 1996).

Though previous findings on the face-specific N170 consistency suggested that the amplitude of the N170 is largest on the posterior temporal areas, and larger on the right brain hemisphere when compared to the left brain hemisphere (Bentin et al., 1996; Webb et al., 2010; Dalrymple et al., 2011), a meta-analysis research revealed that the amplitude of N170 response to facial expressions was significantly affected by the reference electrode (Hinojosa et al., 2015). For example, the effects of facial expression on N170 amplitude were stronger based on AR than that on other references (Rellecke et al., 2013).

Unlike reaction time used in behavior studies, the latency and amplitude of ERP may definitely give us abundant information of what is happening in the brain. Except the amplitude was widely used in N170 studies (Rellecke et al., 2013; Hinojosa et al., 2015), some works also noticed the latency change. A study found that the N170 latency was delayed by inversion of faces, suggesting the extraction of the natural face gestalt was sensitive in brain (Sagiv and Bentin, 2001). A shorter N170 latency was also found in positive faces than that in negative faces, revealing the possible facial feature encoding mechanism (Batty and Taylor, 2003). These results indicated that the N170 latency plays an important role in the study of electrophysiology. However, due to the effect of non-zero reference used in current practice, it is likely that the amplitude and latency of N170 could influence the true neural effect.

The N170 generation involves multiple brain areas such as temporal-parietal regions (Bentin et al., 1996; Itier and Taylor, 2004). Therefore, network analysis may be a more appropriate approach for studying the related neural mechanisms. The time-varying networks based on the adaptive directed transfer function (ADTF) method using a multivariate adaptive autoregressive mode was developed to investigate the time-variant propagation in a simulated electrocorticogram network, which provided consistent results with the cognitive neural science (Li et al., 2016) and clinical assessments performed by neurologists. This could help fully understand the dynamic variation of N170 and uncover more detailed temporal-information processing. In the present study, the time-varying networks of N170 were the first constructed based on the AR and new REST reference (REST) to test the reference effect on the time characteristics in the network levels. Our aim was to evaluate the possible reference effect on not only the current standard ERP analysis such as amplitude and latency, but also the newly information flow from time-varying network analysis on both the real data and the simulation data. Here, simulation was designed to illustrate the reasonability of the real data. We assumed that the time characteristics of REST could be closer to the true case when compared to AR and provide a superior choice for revealing the scalp spatio-temporal changes relating to various cognitive events.

## Materials and methods

### Participants

Thirty normal right-handed male subjects aged from 18 to 25 participated in the experiment. None of them has been reported to have any history of mental or neurological problems. Informed consent was signed prior to the study, and subjects also received a monetary compensation. All experiments were approved by the ethical committee of Chongqing university of Posts and Telecommunications.

### Stimuli and design

A fixation cross (0.5° × 0.5°) was presented at the center of the display throughout the entire block. The stimuli included human face (3° × 3°) and letters (3° × 3°) presented above a fixation cross. Two kinds of stimuli were presented with equal probability in random order. The stimulus onset asynchrony (SOA) varied randomly from 1,000 to 1,200 ms. Stimuli were blocked into sequences of 80 trials, and each subject completed a minimum of 2 blocks. Breaks were permitted between the blocks to maintain a high level of concentration and to prevent fatigue. Subjects were required to fixate the cross and to minimize eye blinks and body motion during all of the experimental blocks. The eye position was monitored with horizontal and vertical electrooculogram (EOG) recordings. Subjects were instructed to make a button-press response with their right index finger to key 1 if face present and key 2 if letter appear, as quickly as possible without making errors.

### Data processing

**Figure 1:**
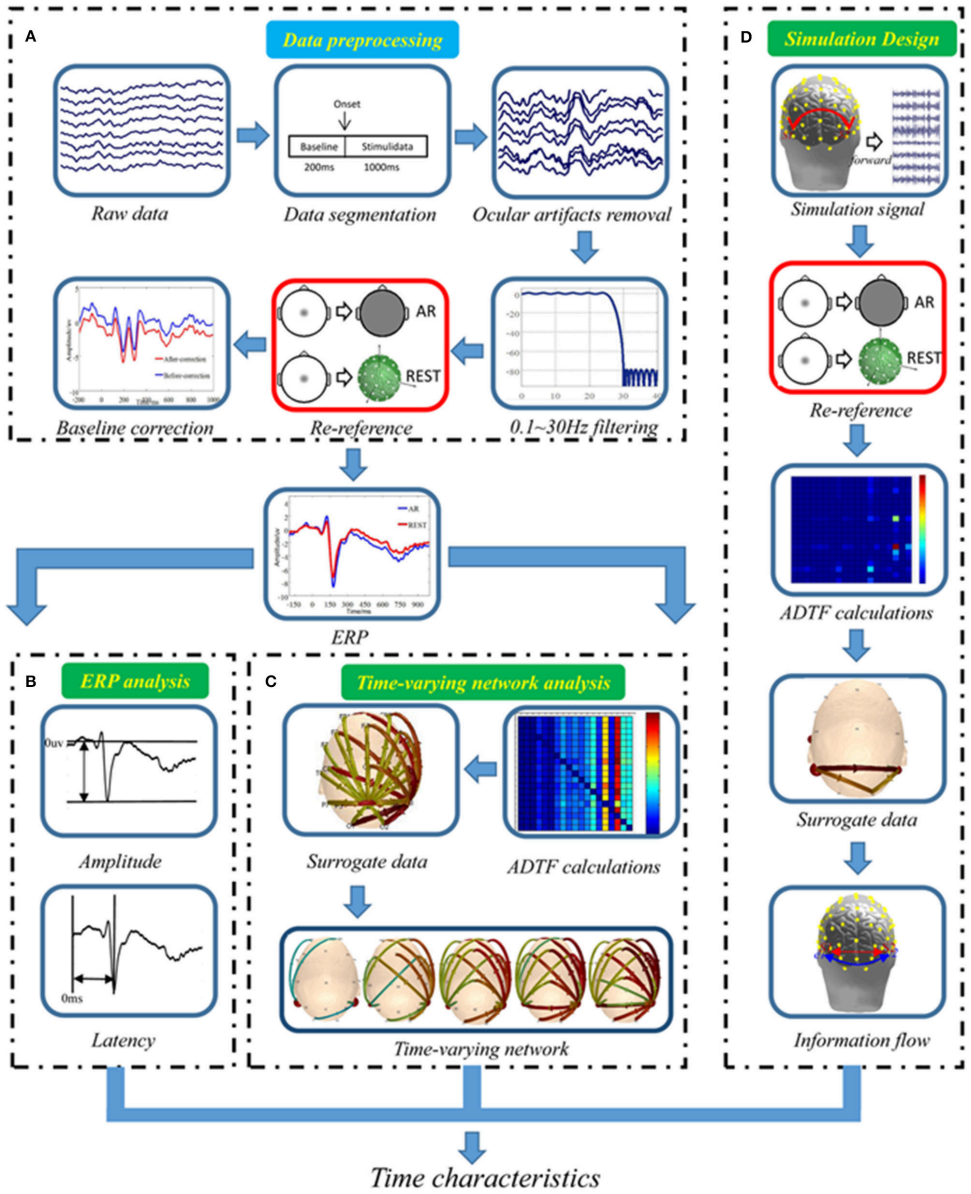
Data analysis procedure. **(A)** Real EEG data preprocessing, **(B)** Standard ERP analysis, **(C)** Real data time-varying network analysis, **(D)** Simulation design.

The data processing included the following four steps: EEG data preprocessing (Figure 1A), ERP analysis (Figure 1B), Time-varying network analysis (Figure 1C) and Stimulation design (Figure 1D).

#### EEG recording and preprocessing (as shown in Figure 1A)

EEG was recorded using a 64-channel NeuroScan system (Quik-Cap, band pass: 0.05–100 Hz, sampling rate: 250 Hz, impedances < 5 kΩ). To monitor ocular movements and eye blinks, EOG signals were simultaneously recorded from four surface electrodes, one pair placed over the higher and lower eyelid and the other pair placed 1 cm lateral to the outer corner of the left and right orbit. Cz was used as the reference during recording online. Then, the EEG was divided into epochs (−200 ms pre- to 1,000 ms post-stimulus onset). Trials with blinks and eye movement were rejected offline on the basis of the EOG. An artifact criterion of ±60 μV was used at all of the other scalp sites to reject trials with excessive electromyographs (EMGs) or other noise transients. The data were re-referenced to the AR (computed as the average of all 64 channels), and REST (the infinity zero reference reconstructed by the software REST, www.neuro.uestc.edu.cn/rest). EEG epochs were sorted according to stimulus types and were averaged from each subject to compute the ERPs. The baseline was defined as the epoch from −200 to 0 ms post-stimulus onset.

#### ERP analysis (as shown in Figure 1B)

Here, we only chose two ERPs, N170 (time window: 160–180 ms) elicited by face stimuli and N1 (time window: 160–180 ms) elicited by letter stimuli, as an example for comparing two references, with respect to amplitude and latency that were induced by the two different stimuli. The amplitudes and the latencies were separately entered into the repeated-measure ANOVAs with two factors: Reference (REST vs. AR) and Stimulus (face vs. letter). Furthermore, the latency difference of N170 between AR and REST were also tested by paired *t*-test. For ANOVA, the partial eta squared $\eta_{p}^{2}$ was used to indicate the magnitude of effect quantities.

#### Time-varying network analysis (as shown in Figure 1C)

To weaken the possible mixture effects such as volume conduction in the current N170 study, the 19 electrodes (i.e., Fp1, Fp2, F7, F3, Fz, F4, F8, T7, C3, Cz, C4, T8, P7, P3, Pz, P4, P8, O1, and O2) of the international 10-20 EEG system were applied in the time-varying network analysis (Xu et al., 2014; Muraja-Murro et al., 2015), which included ADTF calculation, surrogate data, stability evaluation, information flow transfer and statistical analysis (Figure 1C). Details were introduced in the below:

##### ADTF calculation

For each AR and REST related N170 data, after normalization, the multivariate adaptive autoregressive (MVAAR) model was computed by the following equation:

(1) $$\begin{array}{l} X\left(t\right)=\sum_{k=1}^{p}w\left(k,t\right)X\left(t-k\right)+\varepsilon \left(t\right) \end{array}$$

where *X(t)* is the ERP data vector over the entire time window, *w(k,t)* is the coefficients matrix of the time-varying model, which can be calculated by the Kalman filter algorithm, and ε(*t*) represents the multivariate independent white noise. The symbol p denotes the MVAAR model order selected by Schwarz Bayesian Criterion (Schwarz, 1978; Wilke et al., 2008).

After obtaining the MVAAR model coefficient, *w(k,t), H(f,t)* can be obtained from *w(i,t)*, which is then transformed by Equation (2) into the frequency domain. The *H*_*ij*_ element of *H(f,t)* describes the directional information flow between the *j*th and the *i*th element at each time point *t* as:

(2) $$\begin{array}{l} w\left(f,t\right)*X\left(f,t\right)=\varepsilon \left(f,t\right) \end{array}$$

(3) $$\begin{array}{l} X\left(f,t\right)=w^{-1}\left(f,t\right)*\varepsilon \left(f,t\right)=H\left(f,t\right)*\varepsilon \left(f,t\right) \end{array}$$

where $w\left(f,t\right)=\sum_{k=0}^{p}w_{k}\left(t\right)e^{-j2\pi f\Delta tk}$ *w*_k_ is the matrix of the time-varying model coefficients. *w*(*f, t*) and ε(*f, t*) are transforming into the frequency domain as *X(t)* and ε(*t*) respectively.

Defining the directed causal interrelation from the *j*th to the *i*th element, the normalized ADTF is described between (0, 1) as follows,

(4) $$\begin{array}{l} \iota_{ij}^{2}\left(f,t\right)=\frac{|H_{ij}\left(f,t\right){|}^{2}}{\sum_{k}^{n}|H_{ik}\left(f,t\right){|}^{2}} \end{array}$$

To obtain the total information flow from a single node, the integrated ADTF is calculated as the ratio of summation of ADTF values divided by the interested frequency bands [*f1, f2*]:

(5) $$\begin{array}{l} \vartheta_{ij}^{2}\left(t\right)=\frac{\sum_{f1}^{f2}\iota_{ij}^{2}\left(k,t\right)}{f2-f1} \end{array}$$

For N170 ERP signal, the power is mainly concentrated in the 4-10 Hz frequency band (Tang et al., 2008). Hence, we choose to average ADTF values over 4–10 Hz to acquire the final directional information flow for maintaining largest information of N170.

##### Surrogate data

The distribution of ADTF estimator under the null hypothesis of no causal interactions is not well determined, since the ADTF function has a highly non-linear correlation with the time series where it derives. In view of this, the phases of the Fourier coefficients were independently and randomly iterated to produce a new surrogate data, which is a non-parametric statistical test (Wilke et al., 2008). The spectral structure of the time series was retained in the process of iterating the phases of the Fourier coefficients. The shuffling procedure was repeated 200 times for each model-derived time series of each subject in order to establish an empirical distribution of ADTF value under the null hypothesis of no connectivity.

##### Stability evaluation

In accordance with the statistical procedure (Dewan and Rao, 2005), those edges with significant differences determined by the randomly shuffled procedure were chosen by the non-parametric Wilcoxon signed rank test (details see Dewan's group study; Dewan and Rao, 2005). Three significant thresholds, i.e., *p* < 0.05, *p* < 0.03, *p* < 0.01, were set for testing stability difference between the two kinds of time-varying networks constructed via REST and AR. All thresholds were corrected by Bonferroni correction.

The present study mainly focuses on the out-degree weight at the hub electrode site to perform comparative analysis with REST- and AR-based time-varying network under distinct significance levels. Here, the out-degree weight of the *j*th node is defined as the total value of the *j*th column ADTF coefficient matrix in a time-varying network.

##### Information flow transfer

To measure the ability to local information transfer efficiency of the corresponding time-varying networks constructed by the ADTF based on REST and AR, the directed local efficiency based on graph theory was adopted:

(6) $$\begin{array}{l} E_{\vec{loc}}=\frac{1}{2n}\sum_{i\epsilon N}\frac{\sum_{j,m\epsilon N,j\neq i}\left(H_{ij}+H_{ji}\right)\left(H_{im}+H_{mi}\right)\left({\left[d_{\vec{jm}}\left(N_{i}\right)\right]}^{-1}+{\left[d_{\vec{hj}}\left(N_{i}\right)\right]}^{-1}\right)}{\left(k_{i}^{out}+k_{i}^{in}\right)\left(k_{i}^{out}+k_{i}^{in}-1\right)-2\sum_{j\in N}H_{ij}H_{ji}} \end{array}$$

where *H*_*ij*_ is the directed links from j to i and in directed networks. The *H*_*ij*_ does not necessarily equal to *H*_*ji*_. $d_{\vec{hj}}$ denotes the directed shortest path length from h to j. n is the number of all nodes and *N* is the set of all nodes in the network. $k_{i}^{out}=\sum_{j\in N}H_{ji}$, which is the directed out-degree of node *i* and $k_{i}^{in}=\sum_{j\in N}H_{ij}$, that describes the directed in-degree of node *i* in the directed time-varying network.

##### Statistical analysis

Paired *t*-test was performed to measure difference between REST and AR on the out-degree of hub electrode site and the delay of information flow transfer of the time-varying network under the interested time points, respectively. The Cohen's effect size (ES) was further utilized to measure above reference difference. The detailed information about the Cohen's ES can be found in the literature (Fritz et al., 2012).

#### Simulation design

In the present study, the simulation was designed to illustrate the reasonability of the real data analysis. The simulation consisted of three parts: (1) dipole selection and source waveform, (2) the realistic head model for forward computation, and (3) information flow analysis (Figure 1D).

##### Dipole selection and source waveform

**Figure 2:**
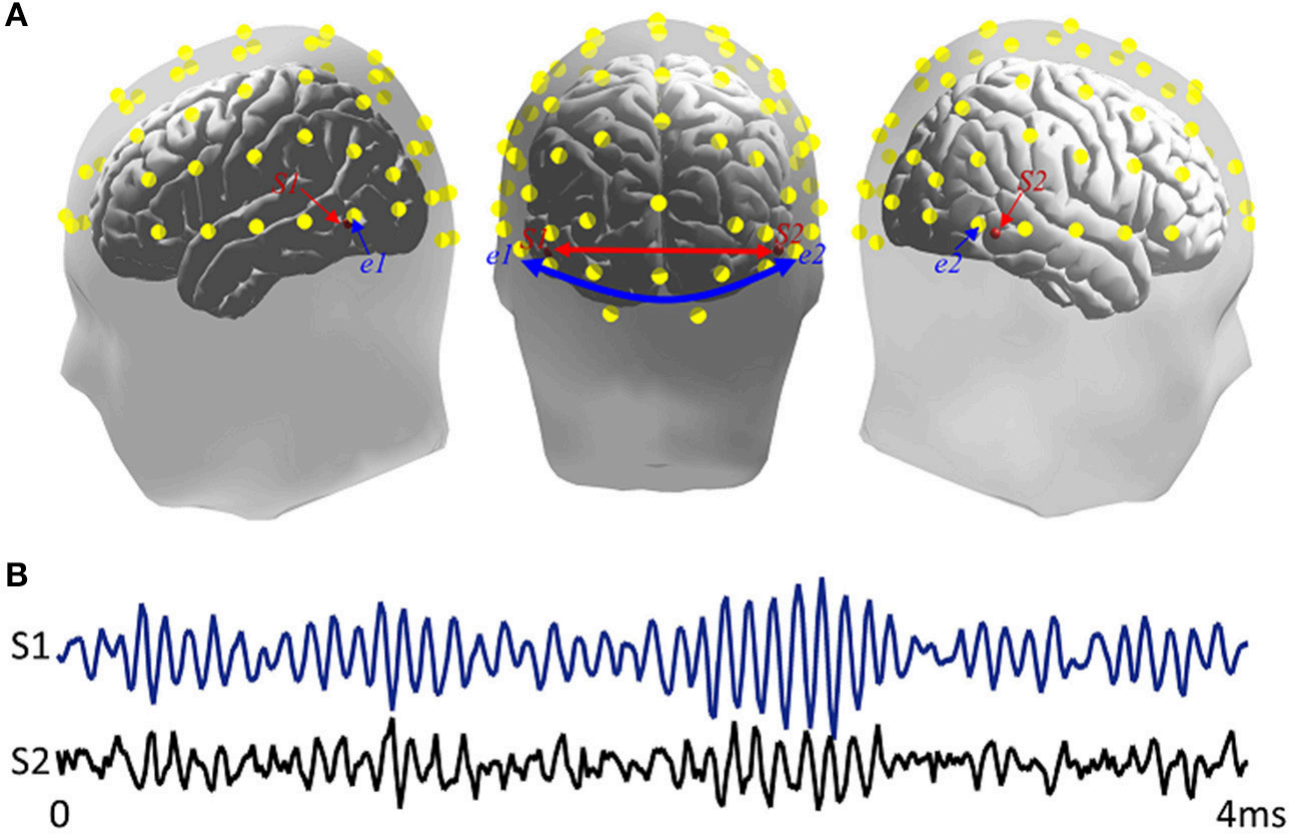
Simulation sources. **(A)** Locations of source and scalp channels in simulation design. S1 and S2 were two dipoles separately distributed in bilateral inferior temporal areas. e1 and e2 presented two scalp electrode channels. The head model comes from Brainstorm anatomy template ICBM512, **(B)** Time series of two simulated signals.

In the simulation, two dipoles, S1 and S2, with fixed orientations at two specified locations with MNI coordinates (−0.57, −0.67, −0.03), and (0.61, −0.63, −0.04), respectively. One dipole was located at the left temporal-parietal region, while the other was located at right temporal-parietal region (Figure 2A). They were regarded as EEG sources in the cortex and had a specific interaction with each other. The forward model was constructed by the Brainstorm toolbox (http://neuroimage.usc.edu/brainstorm/), and time course of dipolar was simulated by the following time-varying casual model Equation (7):

(7) $$\{\begin{array}{c} \text{S}1\left(\text{t}\right)=\text{0}.\text{9}*\text{S}1\left(\text{t}-1\right)-0.9*\text{S}1\left(\text{t}-2\right)+\varepsilon_{1}\left(\text{t}\right)\\ \text{S}2\left(\text{t}\right)=0.5*\text{S}1\left(\text{t}-2\right)+\varepsilon_{2}\left(\text{t}\right) \end{array}$$

Where ε_1_(*t*) and ε_2_(*t*) denoted uncorrelated white noise processes with identical variances. The time courses of S1 and S2 can be seen in Figure 2B.

##### The realistic head model

Here, a 3-shell realistic head model was adopted for EEG forward computation to produce scalp EEG, where conductivities for the cortex, skull, and scalp were 1.0 Ω^−^^1^ m^−^^1^, 1/80 Ω^−^^1^ m^−^^1^, and 1.0 Ω^−^^1^ m^−^^1^, respectively. The solution space was restricted to the cortical gray matter, the hippocampus, and other possible source activity areas, consisting of 15002 cubic mesh voxels with 10 mm inter-distance. The lead field matrix was calculated by the boundary element method (BEM) (Fuchs et al., 1998).

##### Information flow analysis

Time-varying casual model was constructed by using the method of time-varying network analysis as described in the above section and the Brainstorm toolbox as mentioned above. We derived 64-channel spatiotemporally scalp EEG recordings V_AR_ and V_REST_. 19 electrodes array of the international 10-20 system was chosen to decrease the possible effect of volume conduction (Xu et al., 2014; Muraja-Murro et al., 2015). Base on ADTF and surrogate data method, V_S_, V_AR_ and V_REST_ were used to calculated the causal coefficients matrix for time-varying network analysis.

## Results

### ERP measures

**Figure 3:**
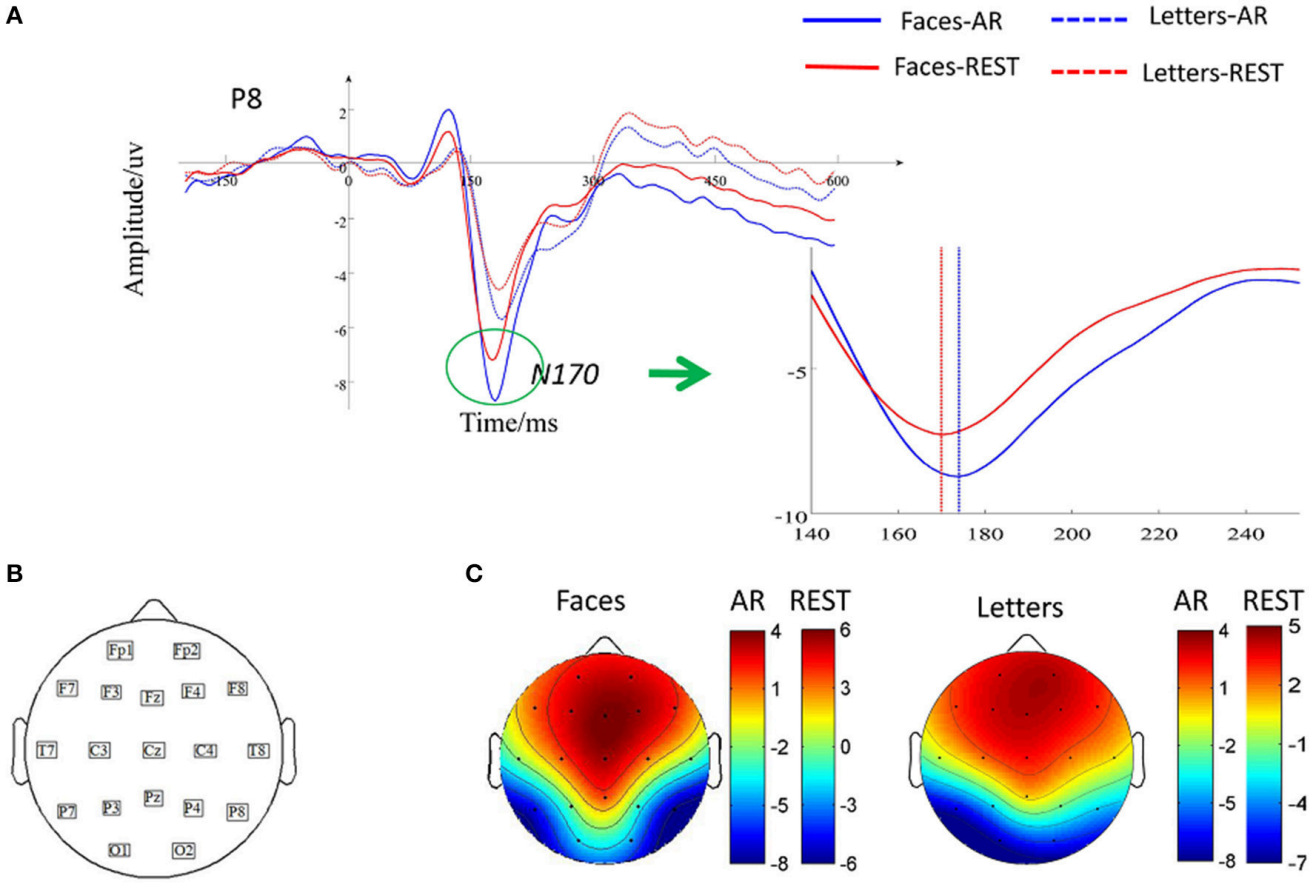
Effects of reference during N170 peak latency and amplitude. **(A)** Grand-averaged ERP for two kinds of stimuli across all subjects at P8. Solid waveform elicited by faces and dashed waveform elicited by letters, **(B)** Distribution of 19 scalp electrodes, **(C)** Topography of ERPs induced by faces and letters in AR and REST.

Figure 3 showed the ERPs elicited by faces and letters based on two different effects. For the N170 amplitude, a repeated-measure ANOVA with two factors (Reference: REST vs. AR; Stimulus: face vs. letter) was performed, significant main effects of reference (*F* = 17.46, *p* < 0.05, $\eta_{p}^{2}=$ 0.17) and stimulus (*F* = 24.79*, p* < 0.05, $\eta_{p}^{2}=$ 0.32) were separately observed. The interaction effect between reference and stimulus was non-significant (*F* = 0.89, *p* > 0.05, $\eta_{p}^{2}=$ 0.003). For the N170 latency, significant main effects of reference (*F* = 4.36, *p* < 0.05, $\eta_{p}^{2}=$ 0.02) and stimulus (*F* = 13.73, *p* < 0.05, $\eta_{p}^{2}=$ 0.25) were separately observed. The interaction effect between reference and stimulus was non-significant (*F* = 2.26, *p* > 0.05, $\eta_{p}^{2}=$ 0.004). Furthermore, a paired *t*-test was performed to measure the reference effect on N170 elicited by human face and the result showed that the latency of N170 at the P8 based on REST was shorter than that on AR [*t* = 4.37, *p* < 0.05, *d* = 0.28; Mean latency±SD for REST: 173.01 ms ±14.69; for AR: 177.60 ms±17.96].

### Time-varying networks in real data

For N170, we constructed the time-varying networks based on AR and REST. The network properties were measured to evaluate the stabilities of the out-degree on hubs and the time characteristics of local efficiency. Furthermore, paired *t*-test and effect size were performance to test the statistical significance of the network properties mentioned above between two references.

#### Stability evaluation

**Figure 4:**
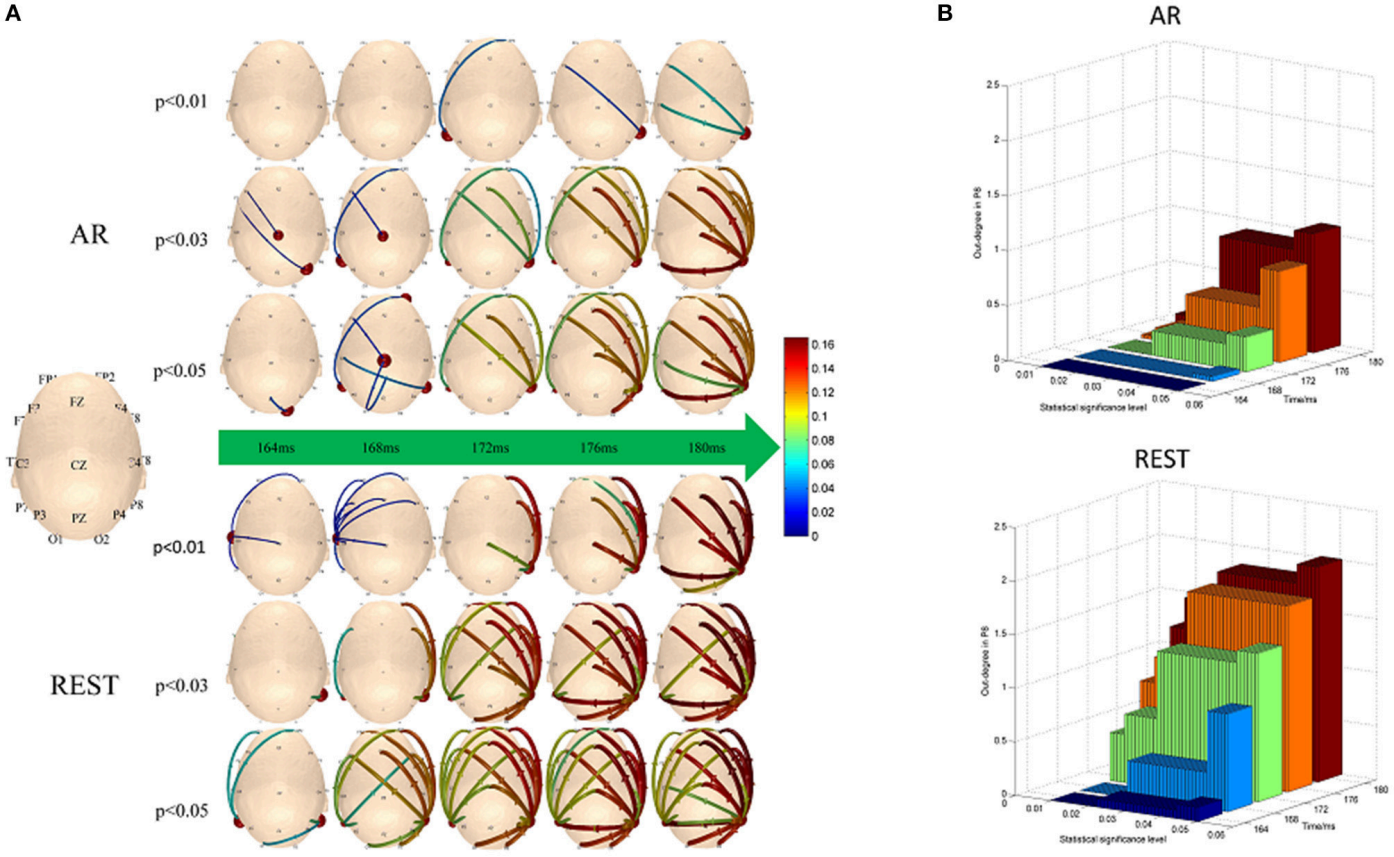
Time-varying networks of N170. **(A)** Hubs and connection mode change over time near 170 ms under the three different significance levels, **(B)** Out-degree of P8.

The hubs and connectivity mode vary with time near 170 ms (from 164 to 180 ms) in AR- and REST-based scalp networks under three thresholds levels (Figure 4). For REST-based scalp time-vary networks, the hubs mainly distributed in the P8 electrode size while the out-degree weight of P8 exhibited few changes at different thresholds levels. However, for AR time-varying networks, the out-degree weight of P8 tended to be zero with the thresholds levels decreasing from 0.05 to 0.01. In contrast, the main pattern was formed earlier when REST used (same as the delay of N170 in Figure 3). More links survived when compared with surrogate test for the thresholds.

#### Time characteristics for local efficiency

**Figure 5:**
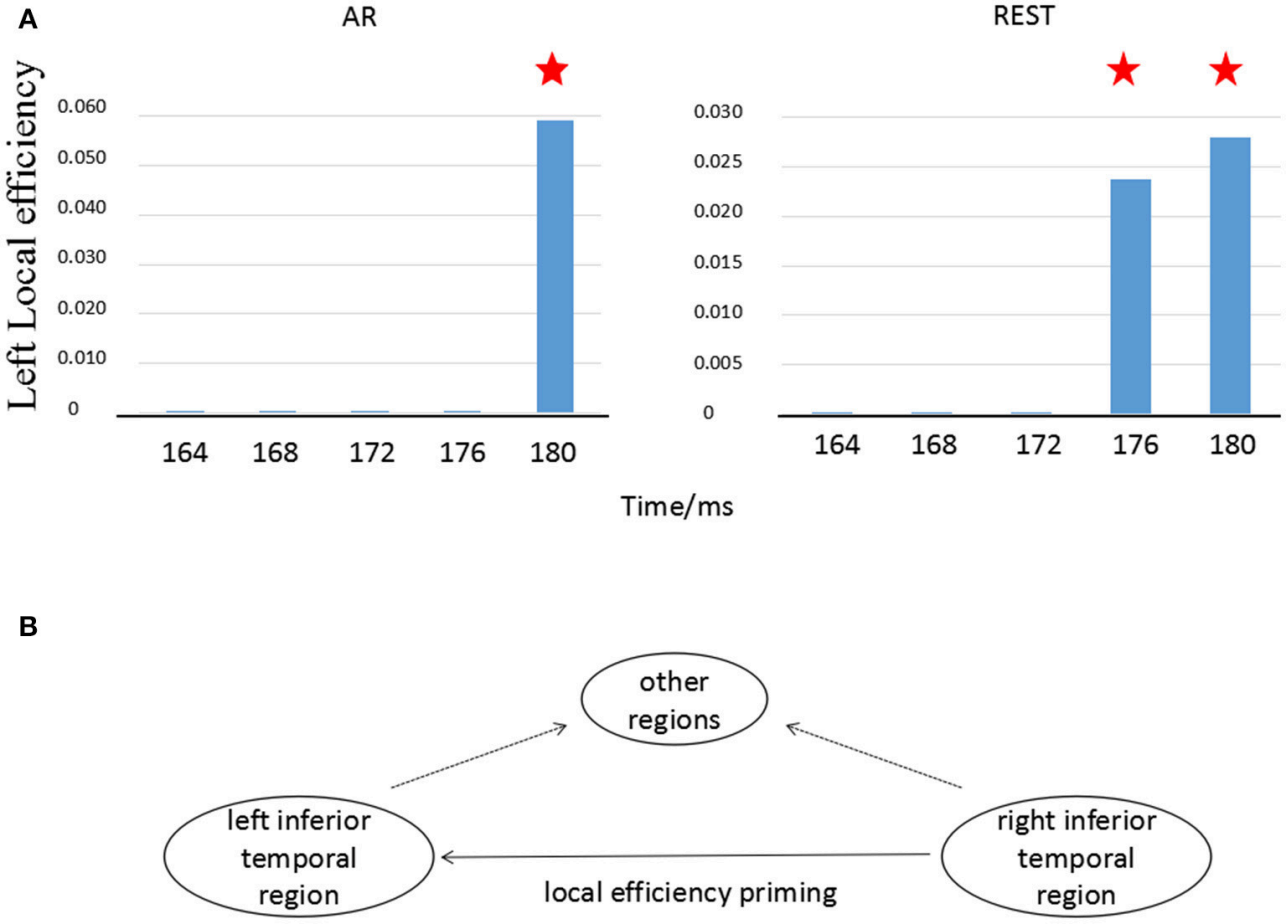
Local efficiency in left temporal-parietal region changes with time. **(A)** Local efficiency changes in left temporal-parietal region over time, **(B)** Illustration of information transfer on local efficiency. The blue bar denotes the transformation of local efficiency in the left temporal-parietal region. The red asterisk (?) represents that there exists an information flow from right temporal-parietal region to left temporal-parietal region.

According to Figure 4, we specially consider the local efficiency in the left temporal-parietal regions, concretely, P7, which was computed via the equation (6). The local efficiency changes over time in left temporal-parietal region (Figure 5A, blue bar), when information flow from right temporal-parietal region to left temporal-parietal region appears (Figure 5A, red asterisk). As shown from Figure 5, it is the appearance of links from the right to left temporal-parietal region that brings about the local efficiency of left temporal-parietal region in the REST network at 176 ms, while the similar phenomenon does not happen until 180 ms in the AR network.

#### Statistical measure

The out-degree of hub node (P8) on N170 based on REST showed greater effect size than that on AR (Table 1). Moreover, the onset delay for information flow transfer from P8 to P7 based on REST was shown to be smaller than that on AR (Table 2).

**Table 1:** Paired *t*-test and effect size for the out-degree of P8 on N170.

**Out-degree of P8**	**Time point (ms)**	***n***	***t***	***p***	***d***
REST>AR	164	30	2.26	<0.05	0.59
REST>AR	168	30	2.18	<0.05	0.52
REST>AR	172	30	2.07	<0.05	0.43
REST>AR	176	30	2.06	<0.05	0.37
REST>AR	180	30	2.06	<0.05	0.32

*n, the number of subjects. d, the effect size*.

**Table 2:** Paired *t*-test and Effect size for the onset delay for P8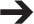 P7 on N170.

**Delay**	***n***	***t***	***p***	***d***
REST < AR	24	−2.53	<0.05	−0.31

*n, the number of subjects. d, the effect size Six subjects were excluded for non-information flow from P8 to P7*.

### Information flow in the simulation case

**Figure 6:**
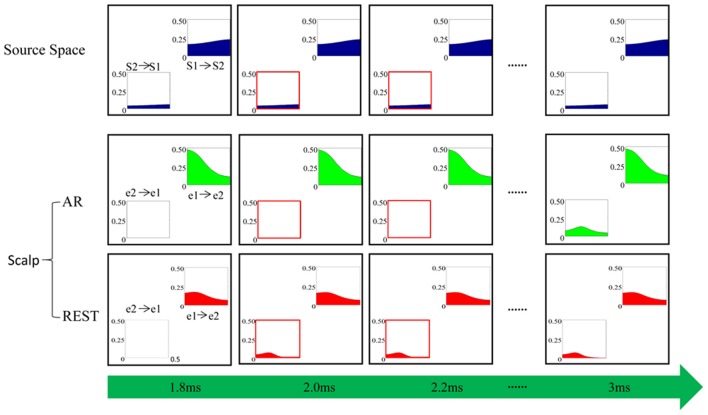
Simulation. “S1-> S2” indicates the causal relationship from source S1 to S2 while “S2->S1” denotes the causal effect of source S2 on S1. Similarly, “e1->e2” is the causal relationship from scalp electrode e1 to e2, and “e2-> e1” denotes the causal influence of e2 on e1. The horizontal axis is normalized frequency and the ordinate represents the causal interconnection values of the corresponding frequency calculated by the ADTF algorithm.

To investigate the time characteristics of two EEG reference methods, i.e., REST and AR, as well as the theoretical possibility of the phenomenon appeared in Figure 5, a simulation experiment was conducted. The results from the time-varying casual model analysis were illustrated in Figure 6. At the given first time point (1.8 ms), there is no causal relationship from scalp electrode e2 to e1 under both AR and REST, while interconnection from S2 to S1 exists in the source space of cerebral cortex, which indicates that connection between paired scalp nodes has certain delay compared to the connection between paired sources in the cortex. At the second time point (2.0 ms), the links from e2 to e1 appear in REST network while the similar relationship does not appear until 3 ms for AR network, illustrating that the scalp information transfer in REST network is earlier than in AR network.

## Discussion

In the present study, two references, AR and REST, were comparatively investigated via the standard analysis of N170 and time-varying networks to reveal the potential effect of different reference on canonical analysis and newly information flow analysis. The main points found in this work were the latency change, and the temporal difference of information flow from right P8 to left P7 electrode when different references were adopted. The stability results showed that the key node distributed at temporal-parietal regions (P8). The REST-based time-varying networks represented a more robust statistical threshold than that of AR-based time-varying networks. The findings via both simulation design and real data illustrated that REST-based networks on scalp showed the time-course closer to that on source space when compared to AR-based time-varying networks on scalp.

### ERPs on different references

In the present study, we separately observed significant main effects on stimulus types and reference as well as non-significant interaction between stimulus and reference for both the amplitude and the latency of ERPs at P8, indicating that the N170 elicited by faces was significantly bigger and earlier than that by letters regardless of REST and AR, which was consistent with the previous studies on N170 (Rellecke et al., 2013; Hinojosa et al., 2015). Moreover, the non-significant interaction illustrated that the difference of ERPs elicited by between faces and letters based on REST was similar to AR. Therefore, face-perception related N170 effects were observed based both on REST and AR through the sample analysis. However, the development of the approximate zero-reference technology would be a timely choice for precise evaluation of the scalp spatio-temporal changes related to various cognitive perceptions. Thus, the choice of correct reference will become an important issue to be addressed. In the present study, we further analyses the reference difference between REST and AR on time-varying network patterns.

### Different stability of hub and connectivity

In the process of establishing statistical network, choosing network statistical threshold is an inevitable step. Different significance levels may induce various network connectivity, leading to confusion in cognitive explanations. Therefore, a less-sensitive method on the aspect of scalp network connectivity threshold is highly desirable. In the present study, REST-related time-varying networks showed stronger adaptability in both hub node (P8) and connectivity strength than that of AR with various thresholds (as shown in Figure 4). For REST, the hubs of the bilateral temporal-parietal regions bias to the right region (P8) were observed in mostly scalp networks during near 170 ms (range from 164 to 180 ms) on different thresholds, which was consistent with the previous converge evidence of N170 ERP studies (Moeller et al., 2008; Johnston and Edmonds, 2009; Dzhelyova et al., 2016). Moreover, the out-degree of P8 appeared to be relative stable regardless of the threshold levels. While for AR, the hubs mutable distributed on the posterior central region (Cz) and the bilateral temporal-parietal regions following with the changes of thresholds. The out-degree of P8 also showed a rapidly decrease when the given thresholds changed from 0.05 to 0.01 (Figure 4B). As shown in Figure A1 of Appendix 2 (Supplementary Material), two cortical connections of the N170 kept strong stability under different threshold levels (i.e., *p* < 0.05, *p* < 0.03, and *p* < 0.01), that is, one connection existed between the right inferior temporal area and the right occipital area in the cortical level, which is consistent with the REST connection between P8 channel and O2 channel in the scalp level. And the other connection existed between bilateral inferior temporal areas in the cortical level, which is consistent with the REST connection between P7 channel and P8 channel in the scalp level. However, AR connections did not appear these two connections observed in the cortical level. Our findings indicated that REST-based scalp varying-networks were superior to AR networks during the interval time about 170 ms which was a crucial time point for the event trigger tasks of face-related detection.

### Different time-course of information flow transfer

Cognitive processing was a multi-stage process due to the interactions of the functions of many brain regions. Previous studies have proved that face recognition resulted from synergistic effects of multiple brain regions including left and right fusiform gyrus (Devue et al., 2007; Hayes et al., 2010). Using functional magnetic resonance imaging (fMRI), researchers found that the representation of facial recognition in the left inferior temporal region depended on the support of the earlier information processing in the right inferior temporal region, suggesting that the information flow transferred from the right brain region to the left region (Verosky and Turk-browne, 2012). According to the fMRI finding, the temporal relationship between bilateral temporal-parietal regions (P7 vs. P8) was tested in the present study. We found that the priming effect of local efficiency at the left temporal-parietal region (P7) was induced by the information flow transfer originally from the right temporal-parietal region (P8) (Figure 5A). This phenomenon was consistent with the conception of local efficiency (Rubinov and Sporns, 2010). In other words, when the information flow from right temporal-parietal region (P8) to left temporal-parietal region (P7) existed, the connection between neighbor nodes of the hub occurred if the hub was deleted (shown in Figure 5B). As shown in Figure 4A, the different time-course on information flow was observed, i.e., the time-course separately 176 ms for REST and 180 ms for AR. The Cohen's ES indicated that the onset delay for information flow from P8 to P7 based on REST was much less than that of AR (Table 2).

### Temporal difference between two references

Though the priming effects of local efficiency were observed in both references, the distinct time-course difference occurred between REST- and AR-based scalp time-varying networks (Figure 5A). The current results showed that for REST, the information transfer efficiency of synergy among multiple brain regions near the left parietal-temporal region was higher than that of AR (Figures 4A, 5A). Recent research reported that the sensitivity of brain could reach sub-millisecond level responding to stimuli (Sperdin et al., 2015), indicating that the temporal difference about 4 ms between REST and AR (Figure 5A) was still a rather valuable time duration for the real brain response.

In the present study, the long temporal difference (4 ms) of information flow transfer between REST and AR (Figure 5) originated from the sampling rate (i.e., 250 Hz). In order to see the theoretical possibility of the temporal effect, a simulation study was conducted in this work. As shown by Figure 6, the time-course of REST-based scalp varying networks was closer to that on source space (Figure 6). Furthermore, the cortical time-varying networks of the N170 [Figure A1 in Appendix 2 (Supplementary Material)] also showed the appearance of the connection from the right to left inferior temporal area was found in 168 ms, which was closer to that of REST when compared to AR. Therefore, AR-based scalp varying networks could induce confusion in the explanation of N170 on temporal characteristics. This clearly suggests that the time-course of information flow for REST could be more accurate than that for AR. And the simulation further supported the time-delay between REST- and AR-based scalp time-varying networks. Further study would be required to confirm the shorter delay difference between these two references.

In general, there are two crucial factors affected scalp network analysis, they are the volume conduction and non-zero reference. For the delay problem here, the conduction from source to scalp is instantaneous, which would not be an issue. However, the mixing effect in AR with signal from all other channels mixed to each channel may change the signal dynamics, and then the latency and the information flow was possibly mislaid. This fact again emphasized the importance to have a true waveform from a zero reference as approximated by REST.

It is noted that coherence or causality has been shown to be influenced by the volume conduction (Xu et al., 2014; Zhang et al., 2015; Li et al., 2016); other techniques such as ICA and LAPLACIAN that are less influenced by the volume conduction effect may be more meaningful for this kind of EEG based network analysis. However, the implementation of them for source estimation may introduce other issues like the component selection of ICA, the effect of high frequency noise on LAPLACIAN (Yao, 2001), which needs to be carefully considered. Therefore, if the time-varying network could be realized on the scalp EEG using sparse electrode array and REST, it may provide the convenience for the corresponding researchers and also weaken the volume conduction effect.

## Conclusion

In this work, the scalp EEG time-varying network was introduced in N170 analysis. It has been revealed that the temporal information flow occurred from right hemisphere to left hemisphere in face recognition of N170. Furthermore, the widely used AR and the newly recommended approximate zero reference (REST) was compared for both the real N170 by using experimental data and the simulation data. It has been demonstrated that AR may induce not only amplitude but also latency change in standard ERP analysis, and a change in information flow in a time-varying network analysis. In addition, our results indicated that REST would be valuable reference for precise analysis of ERP and EEG, which could become a method of choice in various cognitive studies.

## Author contributions

YT: conceived, designed the experiments and Wrote the manuscipt; WX: performed the experiments, analyzed the data and Wrote the first draft; KT: wrote and re-editted the revised manuscipt; HuZ, HaZ, and LY: contributed reagents, materials, analysis tools; ZL and YP: discussed the experiment design, analyzed the data and discussed the experiment results.

### Conflict of interest statement

The authors declare that the research was conducted in the absence of any commercial or financial relationships that could be construed as a potential conflict of interest. The reviewer MLB and handling Editor declared their shared affiliation.
