# Supplementary material for: The Scalp Time-Varying Networks of N170: Reference, Latency, and Information Flow
Source: Front Neurosci. 2018 Apr 18;12:250. doi: 10.3389/fnins.2018.00250 (PMC5915542; doi:10.3389/fnins.2018.00250)
Supplement: Supplementary file 1 [file DataSheet1.docx]

APPENDIX 1

REST：Reference Electrode Standardization Technique

REST is a novel method that builds a bridge between a physical reference and the theoretical neutral reference at an infinity point. For an infinity reference, the forward EEG calculation is given by

$V=GS$ (1)

where G is the transfer matrix referenced at infinity, only dependent on the head model, source configuration, and electrode montage; S is the source; V is the scalp EEG recording with a reference at infinity generated by S. Scalp noise is not explicitly considered in this model. For a physical reference such as the CZ referenced recordings $V_{CZ}$, we similarly have

$V_{CZ}=G_{CZ}S$ (2)

where $G_{CZ}$ is the EEG lead-field matrix with CZ reference and $V_{CZ}$ refers to EEG scalp recordings referenced at CZ. A solution for the source distribution S is given by

$S=G_{CZ}^{-}V_{CZ}$ (3)

where ${(G_{CZ})}^{-}$may be the Moore-Penrose generalized inverse of matrix $G_{CZ}$. From Equations 2 and 3, we can see that the source S is the same, which reflects the fact that reference choice does not influence the source localization; that is, activated neural sources in the brain are not affected by the particular reference used. The potential with reference at infinity can thus be reconstructed as the following:

$V_{REST}=G\left( G_{CZ}^{-}V_{CZ} \right)=UV_{CZ}$ (4)

where $U=GG_{CZ}^{-}$ is the final transfer matrix simultaneously determined by the lead-field matrix G and $G_{CZ}$, where G is known, and $G_{CZ}$can be easily derived from G. In addition, recordings using any other single physical electrode as reference can be mathematically transformed to the infinity reference using a formula similar to

Equation 4; the only difference is the use of a specific lead-field matrix corresponding to the adopted reference.

For more details, please look at the literatures([Yao, 2001](#_ENREF_38); [Qin et al., 2010](#_ENREF_25)) and the website: [www.neuro.uestc.edu.cn/rest](http://www.neuro.uestc.edu.cn/rest) with timely update.

**References**

Qin, Y., Xu, P., and Yao, D. (2010). A comparative study of different references for EEG default mode network: the use of the infinity reference. *Clinical Neurophysiology Official Journal of the International Federation of Clinical Neurophysiology* 121**,** 1981-1991.

Yao, D. (2001). A method to standardize a reference of scalp EEG recordings to a point at infinity. *Physiological Measurement* 22**,** 693-711.

APPENDIX 2

To estimate the cortical activities on N170 signals, we employed the cortically constrained minimum-norm estimate (MNE)([Dale et al., 2000](#_ENREF_5); [Lin et al., 2004](#_ENREF_19)). The MNE were calculated by applying a linear inverse operator W to the measured signals:

$y=Wx$ (1)

where x represented the 19-channel scalp N170 data. y was the corresponding current values in the cortical source space. W was obtained in closed form by minimizing:

$||C^{-1/2}(x-Ay){||}_{2}^{2}+\lambda^{2}||R^{-1/2}y{||}_{2}^{2}$ (2)

Here, C and R denoted covariance matrices of the noise and sources, respectively. A is the gain matrix, that is, the solution of the free source orientation forward problem, thus having three columns for each source location, representing the solution according to each orthogonal direction. $\lambda$ was a regularization parameter([Dale et al., 2000](#_ENREF_5); [Liu et al., 2002](#_ENREF_20)), and $||.||_{2}$ indicated the L2 norm. Minimization of Eq. (2) over y yields

$W=RA^{T}(ARA^{T}+\lambda^{2}C)^{-1}$ (3)

We calculated the regularization parameter by

$\lambda=\frac{trace(ARA^{T})}{trace(C)*SNR^{2}}$ (4)

A fixed value of 5 was used for the signal-to-noise ratio (SNR), which reflected the value in many evoked response experiments.

Here, the 19 sensors were registered to the standard head model with Montreal Neurological Institute (MNI), which consisted of a cortex, skull and scalp with conductivities of 1.0, 1/80, and 1.0, respectively. The forward lead field matrix was calculated via the boundary element method (BEM). Finally, the distribution of the cortical sources related to N170 was estimated.

According to the distribution of scalp electrodes, nineteen cortical regions of interest (ROIs) were selected (Figure A1(A)), and source waveforms of these ROIs were computed by averaging cortical activities within ROIs respectively.

Base on ADTF method and surrogate data (details were shown in session “Real data for time-varying network analysis” in Methods of the present revision), we constructed time-varying networks with three threshold levels (i.e. p<0.05, p<0.03, p<0.01) in cortical space.

Figure A1(B) showed that the hubs and connectivity patterns varied with time from 164ms to 180ms in cortical networks under three thresholds levels. The hubs mainly distributed in the bilateral temporal-parietal regions, the right occipital region and the right frontal region, corresponding to P7, P8, O2, and F8 in the scalp electrode channels, respectively. As shown in Figure A1(C), the out-degree weight of the right temporal-parietal region exhibited stable in under different thresholds levels. These results were similar with our finding in REST, but not appeared in AR.

In the source space, the connection from the right temporal-parietal region to left temporal-parietal region still played an important role to prime the local efficiency of left temporal-parietal region. As shown in Figure A2，the priming time (168ms) of the local efficiency in the left temporal-parietal region, which was close to that of REST when compared to AR. Furthermore, strong connection between the right temporal-parietal region and the occipital region were observed about 170ms, which also be seen in scalp networks with REST, not found in that with AR.


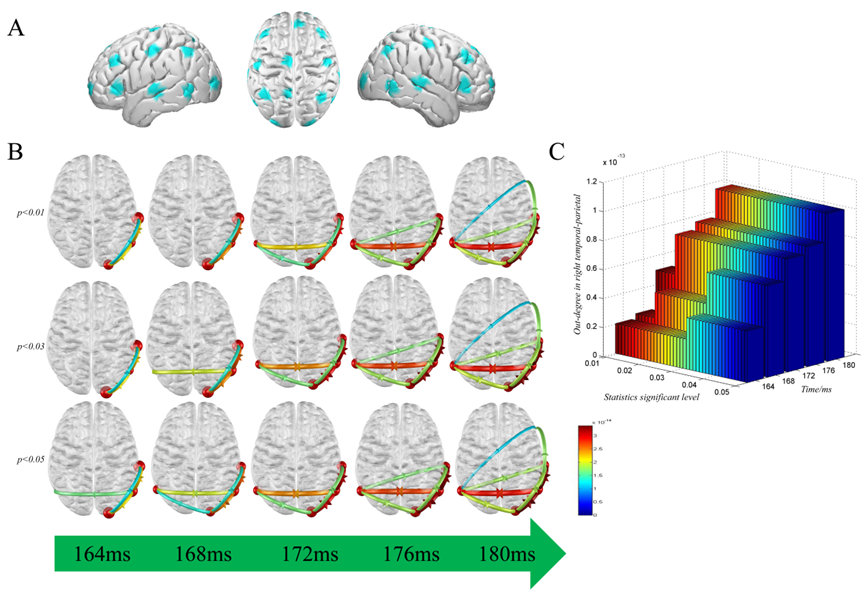


Figure A1. Time-varying networks of N170 in the cortical levels (i.e. source space). (A) The cortical distribution of the 19 selected network nodes. (B) Hubs and connection mode change over time from 164ms to 180ms with three threshold levels. (C) Out-degree of right temporal-parietal nodes.


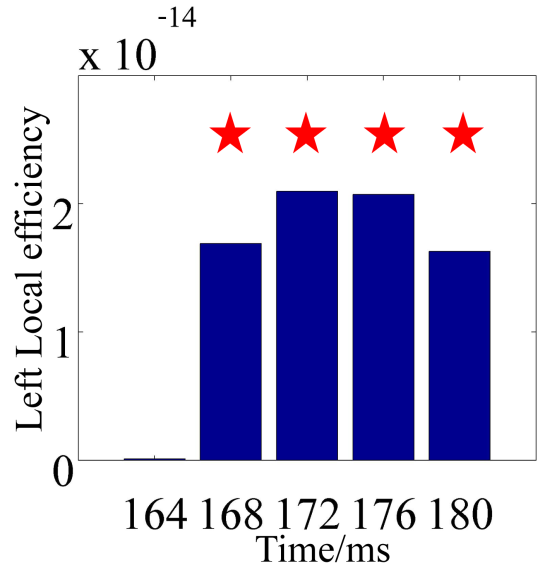


Figure A2. Local efficiency in left temporal-parietal region changes with time

**References**

Dale, A.M., Liu, A.K., Fischl, B.R., Buckner, R.L., Belliveau, J.W., Lewine, J.D., and Halgren, E. (2000). Dynamic statistical parametric mapping: combining fMRI and MEG for high-resolution imaging of cortical activity. *Neuron* 26**,** 55.

Lin, F.H., Witzel, T., Hämäläinen, M.S., Dale, A.M., Belliveau, J.W., and Stufflebeam, S.M. (2004). Spectral spatiotemporal imaging of cortical oscillations and interactions in the human brain. *Neuroimage* 23**,** 582-595.

Liu, A.K., Dale, A.M., and Belliveau, J.W. (2002). Monte Carlo simulation studies of EEG and MEG localization accuracy. *Human Brain Mapping* 16**,** 47.
